# Long Non-Coding RNA AL513318.2 as ceRNA Binding to hsa-miR-26a-5p Upregulates *SLC6A8* Expression and Predicts Poor Prognosis in Non-Small Lung Cancer

**DOI:** 10.3389/fonc.2022.781903

**Published:** 2022-02-16

**Authors:** Yongfei Fan, Yong Zhou, Xinwei Li, Ming Lou, Zhaojia Gao, Kai Yuan, Jichun Tong

**Affiliations:** ^1^ Department of Thoracic Surgery, The Affiliated Changzhou No. 2 People’s Hospital of Nanjing Medical University, Changzhou, China; ^2^ Department of Gastroenterology, Affiliated Cancer Hospital of Bengbu Medical College, Bengbu, China; ^3^ Heart and Lung Disease Laboratory, The Affiliated Changzhou No. 2 People’s Hospital of Nanjing Medical University, Changzhou, China

**Keywords:** competitive endogenous RNA (ceRNA), *SLC6A8*, AL513318.2, hsa-miR-26a-5p, non-small cell lung cancer (NSCLC), prognosis

## Abstract

**Background:**

Studies have demonstrated that the regulatory role of competitive endogenous RNA (ceRNA) networks is closely related to tumorigenesis, which provides new targets for tumor therapy. In this study, the focus was to explore the ceRNA networks that regulate *SLC6A8* expression and their prognosis in non-small cell lung cancer (NSCLC).

**Methods:**

Firstly, the Cancer Genome Atlas (TCGA) data combined with immunohistochemical staining was used to compare *SLC6A8* expression in NSCLC tissues and normal tissues. Thereafter, samples from the immunohistochemical staining of NSCLC were integrated with clinical follow-up data for prognostic analysis. The Starbase database was employed to search for *SLC6A8*-targeted miRNAs and lncRNAs, and survival analysis was performed using clinical data from TCGA to obtain *SLC6A8* expression and prognosis-related ceRNA networks. Finally, the prognostic and therapeutic prospects of *SLC6A8* in NSCLC were further analyzed from methylation sites and the immune microenvironment.

**Results:**

The study results revealed that *SLC6A8* was significantly overexpressed in NSCLC tissues compared to normal tissues, and clinical follow-up data showed that the overexpression group was associated with poor prognosis. In addition, the Starbase data combined with TCGA clinical data analysis demonstrated that the AL513318.2/hsa-miR-26a-5p/*SLC6A8* network regulates *SLC6A8* overexpression in NSCLC and is associated with poor prognosis. Methylation analysis revealed that 11 methylation sites were closely associated with the prognosis of NSCLC. In addition, the immune prognostic risk model showed that the high-risk group was associated with a poorer prognosis than the low-risk group, despite showing a better immunotherapy outcome.

**Conclusion:**

In summary, the AL513318.2/hsa-miR-26a-5p/*SLC6A8* network upregulates *SLC6A8* expression in NSCLC and is associated with poor prognosis. Therefore it may be a prognostic biomarker of NSCLC and a potential therapeutic target.

## Introduction

Lung cancer is one of the top five cancers in humans and has the highest mortality rate of all cancers in the world (1). According to statistics, NSCLC accounts for 85% of patients diagnosed with lung cancer each year, with lung adenocarcinoma (LUAD) and squamous lung cancer (LUSC) subtypes being the most common (2). Although the survival rate of NSCLC patients has improved with the popularization of computed tomography (3), the discovery of new target drugs and the application of immunotherapy (4, 5), their benefits are still not seen in some patients. As we all know, lung cancer results from multiple complex combinations of morphological, molecular, and genetic alterations (6). Tremendous advances in research at the genetic level have been made in regard to NSCLC, such as the discovery of programmed cell death 1 and programmed cell ligand 1 immunotherapeutic targets (7), and the use of long non-coding RNAs (lncRNAs) as biomarkers and therapeutic targets for tumors (8), etc. Therefore, research at the molecular level holds great promise for the treatment of lung cancer.

lncRNAs are a class of non-coding RNAs that are at least 200 nucleotides in length but do not show any protein-coding potential (9). Studies have shown that lncRNAs are one of the important factors affecting cancer development and progression (10), and they can regulate the biological behavior of cancer cells in several ways, such as lncRNA HOTAIR influencing cell growth, migration, invasion, and apoptosis *via* the miR-20a-5p/*HMGA2* axis in breast cancer (11), upregulated LINC01234 promoting NSCLC cell metastasis by activating *VAV3* and repressing *BTG2* expression (12), etc. Therefore, lncRNAs are of great clinical importance as biomarkers of tumor progression.

MicroRNAs (miRNAs) are small single-stranded ncRNAs comprising 19-25 nucleotides, which regulate approximately 30% of the human genome (13) and use mRNAs as binding targets to inhibit gene degradation or translation (14, 15). The proposed hypothesis of ceRNA networks, whereby lncRNAs can competitively bind miRNAs that target mRNAs to regulate mRNA expression (16), further elucidates the regulatory mechanism of cancer development. Currently, ceRNA regulatory networks have been successively reported in various cancers. LINC01133, acting as ceRNA, inhibits gastric cancer progression by sponging miR-106a-3p to regulate *APC* expression and the Wnt/β-catenin pathway (17), while FAM225A promotes nasopharyngeal carcinoma tumorigenesis and metastasis by also acting as ceRNA to sponge miR-590-3p/miR-1275 and upregulate *ITGB3* (18), etc.

The solute carrier family 6 member 8 (*SLC6A8*) encodes a cell surface plasma membrane protein whose function is to transport creatine into and out of cells. Jia Min Loo et al. proposed the idea that extracellular metabolic energetics can promote cancer progression (19). A recent study demonstrated that creatine promotes cancer metastasis through activation of Smad2/3 (20). In addition, a study at the cellular level demonstrated that knockdown of *SLC6A8* significantly induced apoptosis and inhibited migration and invasion of Hep3B and Huh-7 cells in hepatocellular carcinoma cells (21). Therefore, there is a promising research potential regarding *SLC6A8*, however, fewer relevant reports exist in lung cancer. This article focuses on exploring the ceRNA networks that regulate *SLC6A8* expression in NSCLC and its prognosis.

## Materials and Methods

### Transcriptional Level Analysis of *SLC6A8* in NSCLC

First, the “TCGA-ALL-FPKM” dataset (sample=11093) was used to compare the differential expression of *SLC6A8* in tumor tissues and in normal ones in 33 cancers using the Xiantao Academic Online website (https://www.xiantao.love/) (P-value<0.05; wilcoxon rank sum test). Moreover, NSCLC was selected as the study subject, and the transcriptional data of *SLC6A8* in NSCLC (tumor=1037; normal=108) was downloaded from the TCGA database (https://portal.gdc.cancer.gov/). Differential expression of *SLC6A8* in tumor and normal tissues was analyzed using the “limma” R package (P-value<0.05; t-test). Next, the cBioPortal online website (http://www.cbioportal.org/) was used to select 3 LAUD TCGA datasets (TCGA Firehose Legacy; TCGA PanCancer Altas; TCGA Nature 2014) and 3 LUSC TCGA datasets (TCGA Firehose Legacy; TCGA PanCancer Atlas; TCGA Nature 2012) to explore the genetic alterations of *SLC6A8* in NSCLC. Finally, the “polts” section was selected to visualize the relationship between *SLC6A8* expression and a copy number.

### Construction of Tissue Microarrays

Pre-experimental tissue microarrays (TMA) 1 included 57 pairs of NSCLC tissues and paracancerous tissues (18 females and 39 males), purchased from Superbiotek (Shanghai, China). The mean age of patients participating in the study was 61.1 years (range: 34-84 years). (Stage: T1aN0M0 to T4N0M1c; 2004 World Health Organization criteria). TMA1 was mainly used to study paired difference analysis of *SLC6A8* in NSCLC and in paraneoplastic tissues (P-value<0.05; t-test). TMA2 obtained 140 NSCLC tissue samples and 10 normal adjacent tissue samples from patients who underwent surgical resection in the Department of Thoracic Surgery, Zhongshan Hospital, Fudan University, from January 2005 to December 2005. All patients had complete clinical information (38 females and 112 males) and a mean age of 60.1 years (range, 26-79 years) for NSCLC (stage: Ia to IIIa; American Joint Committee on Cancer and the Union for International Cancer Control criteria). Follow-up of this clinical information was recorded until July 2013. TMA2 was used to analyze the unpaired difference expression of *SLC6A8* in NSCLC tissues and normal paraneoplastic tissues (P-value<0.05; t-test). Neither TMA1 nor TMA2 patients received chemotherapy, radiotherapy or biological therapy before surgery.

### Immunohistochemical Staining and Quantification Analysis

To detect the expression of *SLC6A8* in NSCLC, immunohistochemistry was performed using the standard indirect immunoperoxidase procedures. Paraffin specimens were cut 4-µm thick, mounted on slides, baked, deparaffinized and hydrated according to conventional methods. 200 ml of 3% H2O2 and 1 ml of NaN3 were used to inactivate the endogenous peroxidase activity, followed by antigen recovery, which was performed with 10 mM sodium citrate buffer (pH 6.0). Slides were incubated for 1 hour at room temperature in 10 mM TBS with 4% normal rabbit serum (Proteintech) and incubated with primary antibody against *SLC6A8* (1:50, 20299-1-AP, Proteintech Group, Inc, China) at 4°C overnight. Thereafter, the secondary antibody (1:200, K5007, DAKO, China) was developed for 35 minutes at 37°C. Finally, the specimens were weakly re-stained with hematoxylin at 37°C, dehydrated and covered with coverslips.

To quantify the expression of *SLC6A8* protein in NSCLC tissues, the image was converted to grayscale by selecting “8 bit” and the grayscale values were converted to optical density values using “Uncalibrated OD” on ImageJ (22) software. Using the “Set Measurement” module, the area to be stained on the image can be set. The formula for calculating the average optical density (AOD) is as follows:


$$\text{AOD}=\frac{\text{optical density of the stained area}}{\text{stained area}}$$

Paired difference analysis of AOD in tumor tissues and normal tissues in TMA1 was performed using GraphPad Prism software, while unpaired difference analysis was performed in TMA2 (P-value<0.05; t-test). Finally, TMA2 was divided into high- and low-expression groups according to the median AOD value of *SLC6A8*, and the significance of survival between the two groups was analyzed using GraphPad Prism software (P-value<0.05; log-rank test).

### Analysis of Clinicopathological Characteristics

To assess the correlation between *SLC6A8* expression and clinicopathological characteristics, we analysed the correlation between SLC6A8 expression and T stage, N stage, M stage, pathological stage, gender, age and smoking using TCGA data selected from the Xiantao Academic Online website (P-value<0.05). The impact of these clinicopathological factors on overall survival was then analysed by means of univariate and multivariate COX analysis (P-value<0.05).

### Construction of the lncRNA-miRNA-mRNA Triple Regulatory Networks

Firstly, the miRNA data for NSCLC was downloaded from the TCGA database by selecting the “miRNA Expression Qualification” section (cancer=999; normal=91). The miRNA expression matrix was obtained by organizing the raw data using perl software. The Starbase online database (http://starbase.sysu.edu.cn/), a rich database comprised of miRNA-ncRNA, miRNA-mRNA, RBP-RNA and RNA-RNA data that provides a variety of visual interfaces for exploring microRNA targets by searching for microRNA targets through high-throughput CLIP-Seq experimental data and degradome experimental data (23), was then used to find *SLC6A8*-binding miRNAs by selecting “miRNA-mRNA” in the “miRNA-Target” module (programNum>=2). Moreover, the obtained miRNA-mRNA data were subjected to co-expression network construction by Cytoscape software. Since miRNAs and mRNAs are negatively correlated in ceRNA networks, cor<-0.2 and P-value<0.001 were set as correlation filter conditions in order to find *SLC6A8*-related miRNAs in NSCLC. LogFC<0 and diffP-value<0.05 were filtered conditions for differential miRNA expression in tumor tissues and normal tissues.

To explore the targeting relationship between “miRNA-lncRNA”, the screened miRNAs were entered into the “miRNA-Target” module in Starbase, and the screened data were downloaded (programNum>=2). Then, the obtained miRNA-lncRNA data were used for co-expression network construction using Cytoscape software. Since miRNAs and lncRNAs were negatively correlated in the ceRNA network, cor<-0.2 and P-value<0.001 were used as correlation filtering conditions. LogFC>0 and diffP-value<0.05 were used as filtering conditions for differential lncRNA expression in tumor tissues and normal tissues. Finally, the ceRNA networks were obtained by visualizing the ceRNA networks using Cytoscape software.

### Exploring ceRNA Networks With NSCLC-Specific Prognostic Value

Firstly, lncRNA data downloaded in TCGA were used to analyze the correlation of *SLC6A8* with lncRNAs in the regulatory network (cor>0.2, P-value<0.001), and these data were used to further compare the differential expression of lncRNA in NSCLC tissues versus normal tissues (LogFC>0, diffP-value<0.05; t-test). According to the median values of miRNAs and lncRNAs expression in NSCLC, the miRNA and lncRNA samples downloaded from TCGA were divided into high and low expression groups, respectively, and the survival differences between the two groups were analyzed using the log-rank test (P-value< 0.05). Then, the sequences of each lncRNA were queried using the LNCipedia database (https://lncipedia.org/), and the obtained sequences were entered into the lncLocator database (http://www.csbio.sjtu.edu.cn/bioinf/lncLocator/) to obtain the percentage distribution of each lncRNA in the cell. Finally, base pairing between prognosis-related lncRNA-miRNA and mRNA-miRNA were predicted using the Starbase online database.

### Relationship Between Methylation and Expression of *SLC6A8*

First, *SL6A8* methylation levels between LUAD and LUSC tissues and normal tissues were analyzed using the ENSG00000130821 dataset selected from the EWAS Data Hub online database (https://ngdc.cncb.ac.cn/ewas/datahub/index). Thereafter, the association between the expression of *SLC6A8* and their DNA methylation status was investigated using MEXPRESS (https://mexpress.be). Finally, the prognosis of *SLC6A8*-associated methylation sites in NSCLC was analyzed through the EWAS Data Hub online database.

### Immunological Correlation Analysis of *SLC6A8* in NSCLC

The correlation between *SLC6A8* expression and marker gene expression on the surface of six immune cells in NSCLC, the difference between 79 immunomodulators in the high and low *SLC6A8* expression groups and the correlation between 79 immunomodulators and *SLC6A8* expression were first analyzed by the “limma” R package using TCGA data (P-value<0.05). Then, the “survival” R package was used to screen for prognosis-related immunomodulator genes (P-value<0.05). To explore the immuno-prognostic characteristics of *SLC6A8* in NSCLC, we selected prognosis-related immunomodulator genes (cor>0.2) to construct a COX risk proportional regression model. The formula for calculating the risk score was as follows:


$$Risks\;core=\sum_{i=1}^{n}coefi\times Xi$$

The “coefi” and “Xi” represent the coefficient and expression level of each prognosis-related immunomodulator, respectively. Classification of NSCLC patients into high- and low-risk groups was based on median model scores, and a comparison of survival differences between the two groups using the “survivor” and “survminer” R packages (log-rank test; P-value<0.05) was made. Then, a combination of model risk scoring with clinical factors (age, gender, and stage) was made to explore whether risk scoring is an independent prognostic factor for NSCLC. Finally, the efficacy of immunotherapy was assessed between high- and low-risk groups through the Tumor Immune Dysfunction and Exclusion (TIDE) online website. (http://tide.dfci.harvard.edu/).

### Ethical Statement

This study was reviewed and approved by the Research Ethics Committee of The Affiliated Changzhou No. 2 People’s Hospital of Nanjing Medical University. All patients were informed and voluntarily signed an informed consent form for the collection of clinical tissue samples. All specimens were processed and anonymized according to ethical and legal standards.

### Statistics

We implemented all statistical analyses with R (version 4.1.0) and GraphPad Prism 9 (version 9.1.2). The difference in *SLC6A8* expression between the two groups was compared by t test, and the difference in survival between the two groups was compared by log-rank test. P-value<0.05 was considered statistically significant.

## Results

### Expression and Prognosis of *SLC6A8* in NSCLC

**Figure 1:**
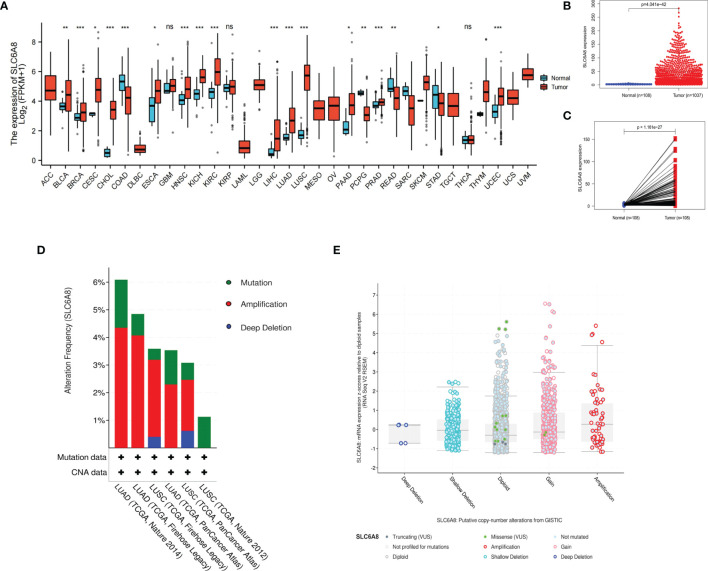
Expression of *SLC6A8* in lung cancer **(A)** Expression of *SLCA8* in pan-cancer *P-value < 0.05; **P-value < 0.01; ***P-value < 0.001. **(B)** Unpaired differential expression and **(C)** paired differential expression of *SLC6A8* in NSCLC in TCGA database. **(D)** Gene alteration and copy number **(E)** analysis of SLCA8 in NSCLC in the eBioPortal online database. ns, no significance.

The results revealed that *SLC6A8* was differentially expressed in various cancers, with *SLC6A8* expression significantly higher in LUAD and LUSC than in normal tissues (**Figure 1A**; P-value<0.05). Analysis of the data downloaded from TCGA showed that *SLC6A8* was significantly overexpressed in NSCLC than in normal tissues (**Figure 1B**; P-value<0.05) or in paraneoplastic ones (**Figure 1C**; P-value<0.05). Genetic alterations analysis results indicated three forms of alterations in *SLC6A8*: amplification, mutation and deep deletion, with gene amplification being the most common, followed by gene mutation (**Figure 1D**). In addition, the results of the copy number analysis also demonstrated that NSCLC samples with the *SLC6A8* amplification exhibited higher mRNA expression (**Figure 1E**).

**Figure 2:**
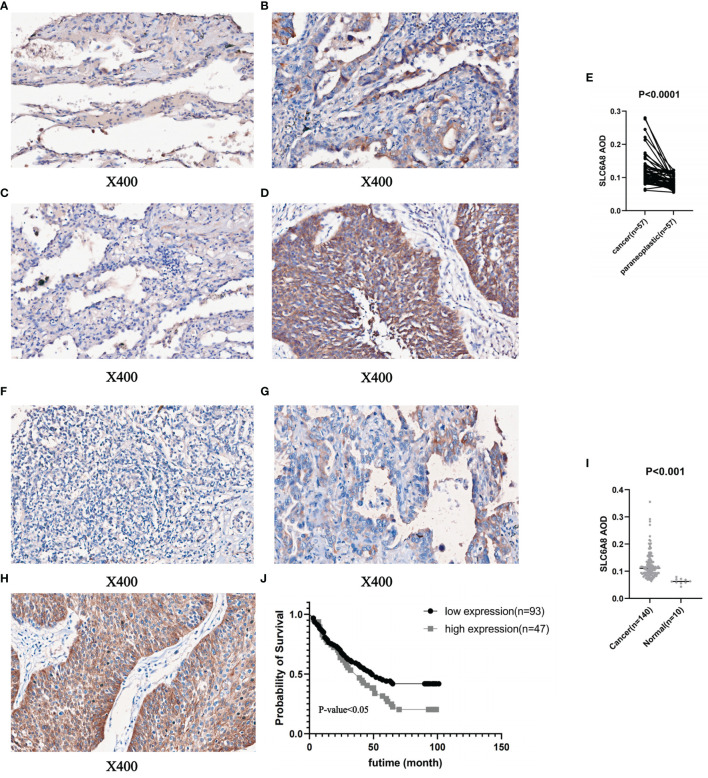
Immunohistochemical staining and quantitative analysis of *SLC6A8* expression in NSCLC. **(E)** Paired differential analysis of *SLC6A8* in IMA1 in NSCLC. **(A)** LUAD paraneoplastic tissue. **(B)** LUAD tissue **(C)** LUSC paraneoplastic tissue **(D)** LUSC tissue **(I)** Unpaired differential analysis of *SLC6A8* in TMA2 in NSCLC **(F)** Normal tissue **(G)** LUAD tissue **(H)** LUSC tissue **(J)** Survival analysis of *SLC6A8* in TMA2 in NSCLC.

To prove the above findings, immunohistochemical staining experiments were performed to analyze the paired difference analysis of *SLC6A8* between 57 pairs of NSCLC and paracancerous tissues and the unpaired difference analysis between 140 tumor samples and 10 normal samples. The results demonstrated that both paired difference and unpaired difference analysis of *SLC6A8* in NSCLC showed statistical significance, and *SLC6A8* expression was significantly higher in tumor tissues than in paraneoplastic (**Figure 2A–E**; P-value<0.05) or normal tissues (**Figure 2F–I**; P-value<0.05). Finally, TMA2 was integrated with data from clinical follow-up and divided into high- and low-expression groups based on the median value of the AOD of *SLC6A8* (0.121), which revealed that the high-expression group showed a poorer prognosis compared to the low-expression group (**Figure 2J**; P-value<0.05).

Analysis of clinicopathological characteristics revealed significant differential expression of *SLC6A8* in T stage, N stage, M stage, pathological stage, gender, age and smoking (**Table 1**; P-value<0.05). In addition, univariate COX analysis showed that T stage (T1&T2 vs. T3&T4), N stage (N0&N1 vs. N2&N3), M stage (M0 vs. M1), pathological stage (Stage I vs. Stage II&III&IV) and age (<=65 vs. >65) were prognostically relevant risk factors. Multivariate COX analysis indicated that these factors could be independent risk factors for prognosis (**Table 2**; P-value<0.05).

**Table 1:** Correlation of SLC6A8 expression with clinicopathological characteristics of NSCLC.

Characteristic	Levels	Low expression of SLC6A8 (n=518)	High expression of SLC6A8 (n=519)	P-value
T stage, n (%)	T1	172 (16.6%)	117 (11.3%)	0.002
	T2	267 (25.8%)	316 (30.6%)	
	T3	55 (5.3%)	65 (6.3%)	
	T4	21 (2%)	21 (2%)	
N stage, n (%)	N0	334 (32.9%)	334 (32.9%)	< 0.001
	N1	93 (9.2%)	133 (13.1%)	
	N2	72 (7.1%)	42 (4.1%)	
	N3	2 (0.2%)	5 (0.5%)	
M stage, n (%)	M0	365 (45.3%)	408 (50.7%)	0.011
	M1	23 (2.9%)	9 (1.1%)	
Pathologic stage, n (%)	Stage I	274 (26.7%)	265 (25.9%)	0.008
	Stage II	124 (12.1%)	161 (15.7%)	
	Stage III	86 (8.4%)	82 (8%)	
	Stage IV	24 (2.3%)	9 (0.9%)	
Gender, n (%)	Female	270 (26%)	147 (14.2%)	< 0.001
	Male	248 (23.9%)	372 (35.9%)	
Age, n (%)	<=65	240 (23.8%)	206 (20.4%)	0.019
	>65	260 (25.8%)	303 (30%)	
Smoker, n (%)	No	73 (7.2%)	20 (2%)	< 0.001
	Yes	429 (42.4%)	489 (48.4%)	

**Table 2:** Univariate and multivariate COX analysis of clinical factors and overall survival in NSCLC.

Characteristics	Total (N)	Univariate analysis		Multivariate analysis	
		HR (95% CI)	P-value	HR (95% CI)	P-value
T stage	1019	1.889 (1.480-2.412)	<0.001	1.366 (1.006-1.854)	0.045
T1&T2	860				
T3&T4	159				
N stage	1000	1.799 (1.372-2.357)	<0.001	1.618 (1.172-2.233)	0.003
N0&N1	882				
N2&N3	118				
M stage	792	2.269 (1.439-3.577)	<0.001	1.733 (1.046-2.870)	0.033
M0	760				
M1	32				
Gender	1022	1.164 (0.949-1.428)	0.145		
Female	410				
Male	612				
Age	1006	1.265 (1.034-1.548)	0.022	1.353 (1.075-1.702)	0.010
<=65	445				
>65	561				
Pathologic stage	1010	1.913 (1.566-2.337)	<0.001	1.364 (1.034-1.799)	0.028
Stage I	533				
Stage II&III&IV	477				
Smoking	996	0.883 (0.617-1.263)	0.496		
No	90				
Yes	906				

### Construction of the lncRNA-miRNA-mRNA Triple Regulatory Networks

**Figure 3:**
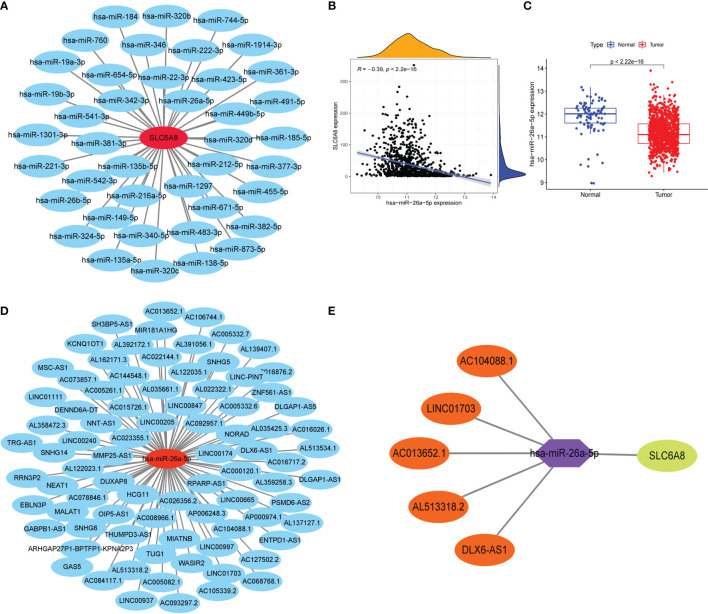
Construction of the lncRNA-miRNA-mRNA triple regulatory network for *SLC6A8* overexpression in NSCLC. **(A)** Starbase database of miRNAs targeting binding to *SLC6A*8 in NSCLC **(B, C)**Targeted miRNAs associated with *SLC6A8* expression in a triple-regulatory network (cor<-0.3, P-value < 0.001, logFC<0. diffP-value < 0.05 ) **(D)** Starbase database of IncRNAs targeting binding to hsa-miR-26a-5p in NSCLC. **(E)** The ceRNA regulatory network of *SLC6A8* overt expression in NSCLC.

To explore the lncRNA-miRNA-mRNA networks that regulate the expression of *SLC6A8* in NSCLC, the expression matrix of miRNAs from the TCGA database combined with *SLC6A8*-related miRNAs from the Starbase database (miRNA=41) was extracted to construct a miRNA-mRNA co-expression network (**Figure 3A**). In the miRNA-mRNA correlation analysis, there was a correlation between hsa-miR-26a-5p expression and *SLC6A8* expression in NSCLC (cor<-0.2, P-value<0.001; **Figure 3B**), and hsa-miR-26a-5p expression was significantly lower in NSCLC than in normal tissues (P-value<0.05; **Figure 3C**). Analysis using the Starbase database identified 84 target lncRNAs (lncRNA=97) associated with hsa-miR-26a-5p (**Figure 3D**). Among them, LINC01703, AC104088.1, DLX6-AS1, AC013652.1 and AL513318.2 met the filtering conditions for correlation analysis with hsa-miR-26a-5p (cor<-0.2, P-value<0.001), and they were significantly overexpressed in NSCLC than in normal tissues (logFC<0, diffP-value<0.05) (**Table 3**). Finally, the ceRNA networks that regulate *SLC6A8* expression in NSCLC were mapped using Cytoscape software (**Figure 3E**).

**Table 3:** Targeted lncRNAs in the ceRNA network associated with hsa-miR-26a-5p and *SLC6A8* expression.

lncRNA	hsa-miR-26a-5p (cor)	*SLC6A8 *(cor)	P-value	logFC	diffPval
LINC01703	-0.2026	0.327	1.34E-10	1.149	1.02E-54
AC104088.1	-0.2834	0.5713	1.09E-19	0.506	6.65E-16
DLX6-AS1	-0.271	0.5635	4.48E-18	0.3243	6.00E-34
AC013652.1	-0.2155	0.5437	7.86E-12	0.6087	8.98E-34
AL513318.2	-0.2278	0.5244	4.34E-13	1.966	0.0008941

### Prognosis-Related ceRNA Regulatory Networks of *SLC6A8* in NSCLC

**Figure 4:**
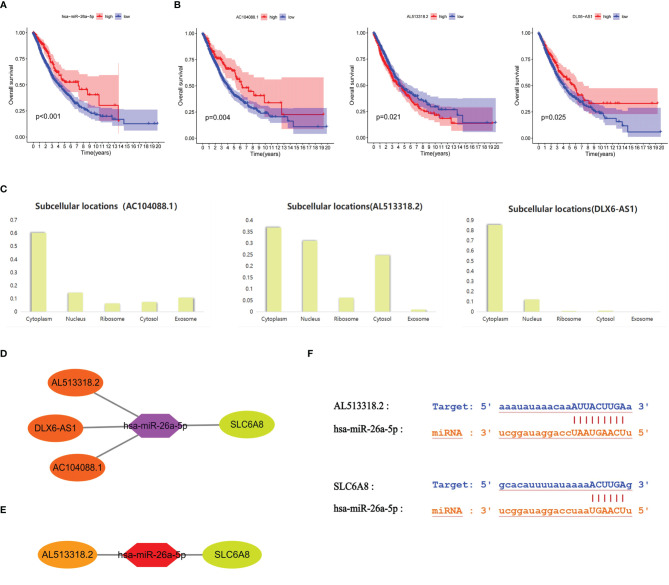
Prognostic ceRNA regulatory network associated with *SLC6A8* overexpression in NSCLC **(A)** Prognostic miRNAs associated with *SLC6A8* overexpression in NSCLC **(B)** Prognostic IncRNAs associated with *SLC648* overexpression in NSCLC **(C)**. Localization of prognostic IncRNAs in NSCLC cells **(D)**. The ceRNA network regulating *SLC6A8* overexpression and prognosis in NSCLC **(E)**. The ceRNA network regulating *SLC6A*8 overexpression and poor prognosis in NSCLC **(F)** Base pairing between Al 513318.2/hsa-miR-26a-5p and *SLC6A8*/has-miR-26a-5p in Starbase online database.

Survival analysis revealed that the low expression group of hsa-miR-26a-5p in NSCLC had a poorer prognosis compared to the high expression group (cut point=11.68; **Figure 4A**). Meanwhile, hsa-miR-26a-5p was significantly less expressed in NSCLC tissues than in normal tissues (**Figure 3C**), so hsa-miR-26a-5p could be used as a prognostic biomarker for NSCLC. The study demonstrated that LINC01703, AC104088.1, DLX6-AS1, AC013652.1 and AL513318.2 were significantly associated with *SLC6A8* (cor>0.2, P-value<0.001) and were significantly differentially expressed in NSCLC tissues versus normal tissues (logFC<0, P-value< 0.05) (**Table 3**). lncRNAs survival analysis showed that the AC104088.1 (cut point=1.172) and DLX6-AS1 (cut point=0.4318) low expression groups had a poor prognosis compared to the high expression group, while the AL513318.2 (cut point=0.5129) high expression group was associated with a poor prognosis (**Figure 4B**). Cellular localization analysis showed that AC104088.1, DLX6-AS1 and AL513318.2 had the highest percentage in the cytoplasm (**Figure 4C**). Therefore, AC104088.1, DLX6-AS1 and AL513318.2 can be used as prognostic biomarkers for NSCLC (**Figure 4D**). Since SLC6A8 is highly expressed in NSCLC and associated with a poor prognosis, the AL513318.2/hsa-miR-26a-5p/*SLC6A8* regulatory network could be used as a biomarker for poor prognosis and a new target for the treatment of NSCLC (**Figure 4E**). In addition, base pairing between AL513318.2/hsa-miR-26a-5p and *SLC6A8/*hsa-miR-26a-5p were predicted using the Starbase online database (**Figure 4F**).

### Correlation Between Methylation and *SLC6A8* Expression

**Figure 5:**
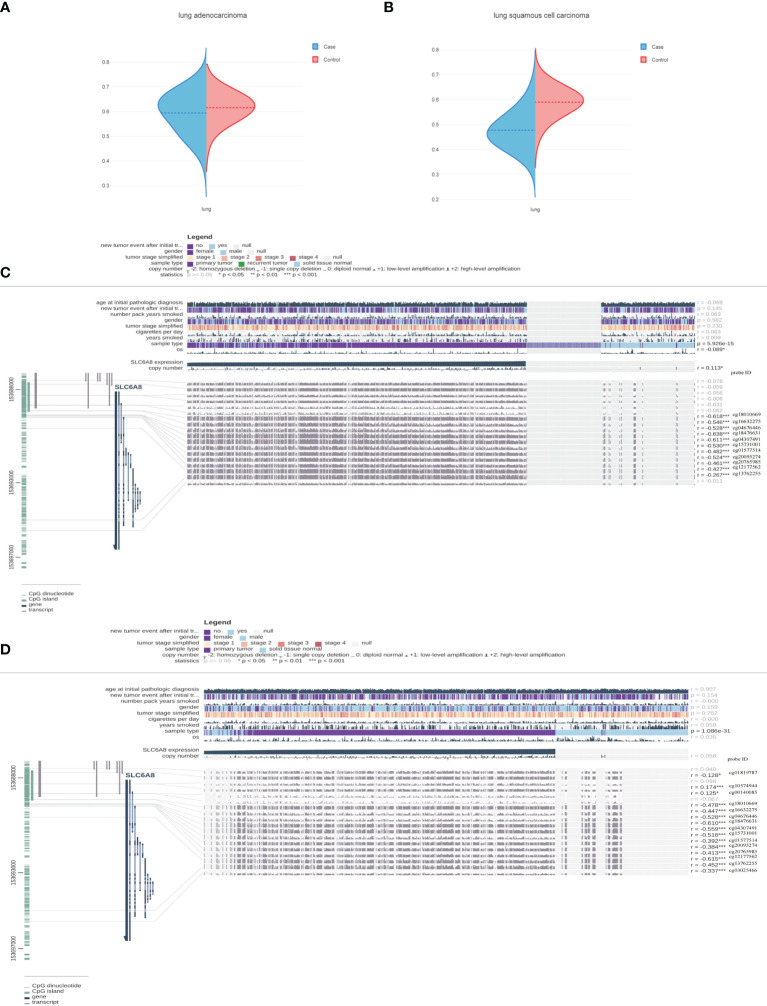
Methylation sites associated with *SLC6A8* expression in NSCLC Methylation levels associated with *SLC6A8* expression in LUAD **(A)** and LUSC **(B)** compared to normal tissues. Methylation regulatory sites associated with *SLC6A8* expression in LUAD **(C)** and LUSC **(D)**. (r<-0.2; *P-value < 0.05; **P-value < 0.01; ***P-value < 0.001).

To further explain the mechanism of aberrant upregulation of *SLC6A8* in NSCLC tissues, the correlation between *SLC6A8* expression levels and its methylation status was explored. Analysis of the ENSG00000130821 dataset in the EWAS Data Hub online database showed that methylation levels in LUAD (**Figure 5A**; **Supplementary Material 1**) and LUSC (**Figure 5B**; **Supplementary Material 2**) negatively correlated with *SLC6A8* expression, and the methylation levels in tumor tissues were lower than those in normal tissues. Then, the MEXPRESS database exhibited multiple methylation regulatory sites in LUAD (**Figure 5C**) and LUSC (**Figure 5D**) that were negatively associated with *SLC6A8* expression (r<-0.2, P-value<0.05). Among them, cg18010669, cg16632275, cg04676446, cg18476631, cg04307491, cg15731001, cg01577514, cg20095274, cg20765985, cg12177562 and cg13762255 are involved in *SLC6A8* expression regulation in both LUAD and LUSC. Methylation site survival analysis indicated that 11 methylation sites expression were significantly associated with LUAD and LUSC survival (**Supplementary Material 3**). In addition, cg12177562, cg13762255 and cg20765985 methylation sites had significantly poorer OS in the low expression group compared to the high expression group in both lung adenocarcinoma and lung squamous carcinoma.As these sites were significantly negatively correlated with *SLC6A8* expression in lung adenocarcinoma and lung squamous carcinoma, these three sites may be potential therapeutic targets for modulating the poor prognosis of *SLC6A8* in NSCLC.

### Immunological Role of *SLC6A8* in NSCLC

**Figure 6:**
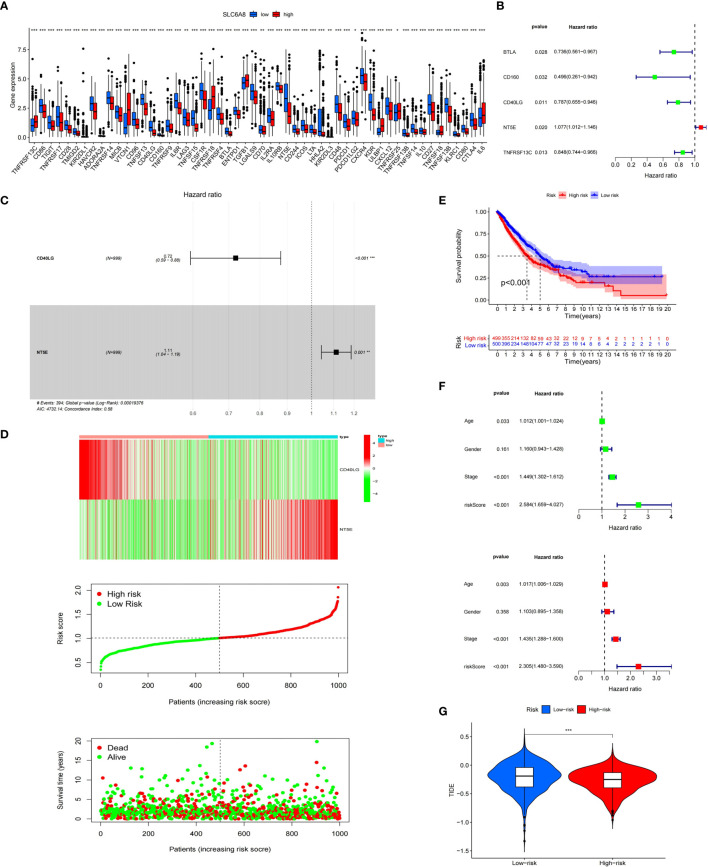
Immunological correlation analysis of *SLC6A8* in NSCLC **(A)** Differential analysis of immunomodulators between high and low *SLC6A8* expression groups in NSCLC **(B)** Prognostic analysis of immunomodulators associated with *SLC6A8* in NSCLC **(C, D)** *SLC6A8*-related immune prognostic risk model in NSCLC **(E)** Prognostic differences between high and low risk groups in the risk model **(F)**. Model risk scoring combined with clinical factors for univariate and multivariate COX analysis **(G)**. Comparison of immunotherapy effects between high and low risk groups of the risk model. (*P-value < 0.05; **P-value < 0.01, ***Pvalue < 0.001.

Immunological correlation analysis showed that *SLC6A8* expression mainly correlated negatively with B cell, CD8+ T cell, M1 macrophage, M2 macrophage, Neutrophil and Dendritic cell surface marker genes, with the most negative correlation with *NRP1* (**Table 4**). Research indicated that immunomodulators are an important component of the tumor immune microenvironment, and their alterations are closely related to the prognosis of patients (24, 25). In this study, 54 of the 79 immunomodulators had significant differences between the high and low *SLC6A8* expression groups (**Figure 6A**), of which 31 immunomodulators predominantly correlated negatively with *SLC6A8* expression, with *CD40LG* being the most negatively correlated (**Table 5**). Then, prognostic analysis of 31 *SLC6A8*-associated immunomodulators in NSCLC indicated *BLTA*, *CD160*, *CD40LG*, and *TNFRSF13C* as low-risk factors and *NT5E* as high-risk factors (**Figure 6B**). To explore *SLC6A8*-mediated immune prognosis, risk proportional regression models of *SLC6A8* prognosis-associated immunomodulator genes (**Figures 6C, D**) were constructed. The model risk scoring formula is as follows: risk core = (coefficient × Expression *CD40LG*) + (coefficient × Expression *NT5E*) The high-risk group classified according to the median value (1.024) of the model risk scoring had poorer survival compared to the low-risk group (**Figure 6E**). Moreover, model risk scoring combined with clinical factors in univariate and multifactorial COX analyses suggested that age, stage and risk scoring could be independent risk factors for NSCLC prognosis (**Figure 6F**). Finally, the effect of immunotherapy was evaluated between the high- and low-risk groups using the TIDE database, and the results revealed that immunotherapy was significantly better in the high-risk group than in the low-risk group (**Figure 6G**).

**Table 4:** Correlation analysis of *SLC6A8* expression and immune cell surface marker genes.

Immune Cell	gene	cor	pvalue
B cell	*CD19*	-0.1427	**4.06E-06**
	*CD79A*	-0.1342	**1.47E-05**
CD8+ T cell	*CD8A*	-0.2347	**2.29E-14**
	*CD8B*	-0.1549	**5.36E-07**
	*CD4*	-0.3972	**2.2E-16**
M1 macrophage	*NOS2*	0.3501	**4.06E-06**
	*IRF5*	0.2148	**1.47E-05**
	*PTGS2*	-0.05677	**2.29E-14**
M2 macrophage	*CD163*	-0.3125	**2.2E-16**
	*VSIG4*	-0.3595	**2.2E-16**
	*MS4A4A*	-0.3609	**2.2E-16**
Neutrophil	*CEACAM8*	-0.4486	**1.77E-52**
	*ITGAM*	-0.3253	**2.2E-16**
	*CCR7*	-0.3144	**2.2E-16**
Dendritic cell	*HLA-DPB1*	-0.5229	**2.2E-16**
	*HLA-DQB1*	-0.4677	**2.2E-16**
	*HLA-DRA*	-0.5093	**2.2E-16**
	*HLA-DPA1*	-0.5159	**1.36E-71**
	*CD1C*	-0.4303	**2.2E-16**
	*NRP1*	-0.5868	**2.2E-16**
	*ITGAX*	-0.2268	**1.70E-13**

The significance of the bolded values is P-value < 0.05.

**Table 5:** Correlation analysis of SLC6A8 expression with immunomodulators.

gene	cor	pvalue
*ADORA2A*	-0.1763	**1.09E-08**
*BTLA*	-0.3565	**2.20E-16**
*CD160*	-0.3286	**2.20E-16**
*CD244*	-0.3139	**2.20E-16**
*CD27*	-0.2109	**7.81E-12**
*CD28*	-0.3497	**2.20E-16**
*CD40LG*	-0.5052	**2.89E-68**
*CD48*	-0.3265	**2.20E-16**
*CD70*	-0.0964	**0.001885**
*CD80*	-0.3335	**2.20E-16**
*CD86*	-0.3102	**2.20E-16**
*CD96*	-0.3107	**2.20E-16**
*CSF1R*	-0.3592	**2.20E-16**
*CTLA4*	-0.2609	**1.75E-17**
*CXCL12*	-0.2074	**1.73E-11**
*CXCR4*	-0.2534	**1.51E-16**
*ENTPD1*	-0.3223	**2.20E-16**
*HAVCR2*	-0.3463	**2.20E-16**
*HHLA2*	-0.2718	**5.14E-19**
*ICOS*	-0.2845	**1.12E-20**
*IL10*	-0.1981	**1.34E-10**
*IL10RB*	-0.2422	**3.26E-15**
*IL2RA*	-0.1842	**2.46E-09**
*IL6*	0.1512	**1.03E-06**
*IL6R*	-0.4263	**2.20E-16**
*KDR*	-0.5113	**2.20E-16**
*KIR2DL1*	-0.09579	**0.002015**
*KIR2DL3*	-0.1022	**0.0009831**
*KLRC1*	-0.2163	**1.93E-12**
*LGALS9*	-0.214	**3.80E-12**
*LTA*	-0.2548	**1.02E-16**
*MICB*	-0.1768	**1.05E-08**
*NT5E*	-0.4325	**2.20E-16**
*PDCD1*	-0.1944	**2.96E-10**
*PDCD1LG2*	-0.08201	**0.008252**
*TGFB1*	0.1714	**2.93E-08**
*TIGIT*	-0.2082	**1.43E-11**
*TMIGD2*	-0.2498	**3.19E-16**
*TNFRSF13B*	-0.2873	**3.74E-21**
*TNFRSF13C*	0.2205	**8.06E-13**
*TNFRSF14*	-0.3083	**2.20E-16**
*TNFRSF17*	-0.1988	**1.06E-10**
*TNFRSF18*	0.4321	**2.05E-48**
*TNFRSF25*	0.1097	**0.0004067**
*TNFRSF4*	-0.2370	**1.28E-14**
*TNFRSF9*	-0.1931	**3.89E-10**
*TNFSF13*	-0.5166	**2.20E-16**
*TNFSF13B*	-0.4497	**2.20E-16**
*TNFSF14*	-0.3529	**2.20E-16**
*TNFSF15*	-0.5273	**2.20E-16**
*TNFSF18*	0.1279	**3.62E-05**
*ULBP1*	0.4382	**6.85E-50**
*VTCN1*	0.3910	**3.24E-39**

The significance of the bolded values is P-value < 0.05.

## Discussion

Because of the insidious early symptoms and highly aggressive biology of lung cancer, most patients are at advanced stages upon diagnosis. Although the application of new clinical diagnostic techniques, such as computed tomography (26), tumor markers (27), genetic testing (28), etc., has improved lung cancer patients’ survival rate, the mortality rate still ranks the highest of all cancers. Therefore, exploring the regulatory mechanisms of lung cancer development and finding new effective biomarkers is crucial for the diagnosis and prognosis of patients. In this study, we distinguish from the usual biomarkers, such as individual genes, individual lncRNAs, etc., by establishing a triple regulatory network of lncRNA-miRNA-mRNA associated with *SLC6A8* expression and linking it to patients’ prognosis. Currently, the regulatory role of ceRNA in lung cancer is being explored. For example, lncRNA LCAT1 acts as a ceRNA for miR-4715-5p, leading to the upregulation of GTPase 1 activity, which affects the proliferation, invasion and migration of lung cancer (29).

The main finding of this study was identified a prognosis-related ceRNA regulatory network (AL513318.2/hsa-miR-26a-5p/*SLC6A8*) in NSCLC. In the ceRNA regulatory network, hsa-miR-26a-5p was significantly negatively correlated with *SLC6A8* expression, and AL513318.2 was significantly negatively correlated with hsa-miR-26a-5p expression, while significantly positively correlated with *SLC6A8* expression. The authenticity of the ceRNA regulatory axis was confirmed by base complementary pairing. In addition, AL513318.2 and *SLC6A8* were significantly overexpression in NSCLC tissues compared to normal tissues, and survival analysis revealed that the high expression group had a poorer prognosis compared to the low expression group, whereas hsa-miR-26a-5p exhibited low expression in NSCLC tissues compared to normal tissues, and survival analysis revealed that the low expression group had a poorer prognosis compared to the high expression group. These results consistently suggest that AL513318.2/hsa-miR-26a-5p/*SLC6A8* is a poor prognosis-associated ceRNA regulatory network in NSCLC. The advantages of our ceRNA regulatory network compared to genes or lncRNAs is that each target has survival significance and either individual targets or the ceRNA network can be used as prognostic biomarkers. In addition, the ceRNA network we constructed could also elucidate the regulatory mechanism of *SLC6A8* overexpression and poor prognosis in NSCLC. Therefore, the ceRNA regulatory network we constructed, AL513318.2/hsa-miR-26a-5p/*SLC6A8*, is more effective than single genes or lncRNAs.

Xianzheng Qin et al. revealed that hsa-miR-26a-5p can be used as a potential prognostic biomarker for patients with intrahepatic cholangiocarcinoma (30). Moreover, hsa-miR-26a-5p was proposed to be associated with the prognosis of gastrointestinal tumors by Zheng Chen et al (31). However, there are no relevant reports on the functional and mechanistic roles of AL513318.2 until now. Our study provides the first evidence that AL513318.2 is highly expressed in NSCLC and negatively correlates with hsa-miR-26a-5p expression to regulate poor prognosis of SLC6A8 in NSCLC. A total of five lncRNAs (LINC01703, AC104088.1, DLX6-AS1, AC013652.1 and AL513318.2) were found to be significantly negatively correlated with hsa-miR-26a-5p expression and positively correlated with *SLC6A8* expression in this study. Those with survival significance included AL513318.2, AC104088.1 and DLX6-AS1, where the only ceRNA network satisfying a poor prognosis was AL513318.2/hsa-miR-26a-5p/*SLC6A8*, while AC104088.1 and DLX6-AS1 showed the opposite prognosis. So what are the reasons for this opposite phenomenon? Firstly, it has been demonstrated that LncRNAs can act as tumor suppressor genes and oncogenes transcriptional regulators (8, 32). High expression of some oncogenes in tumors that exhibit poor prognosis also leads to feedback high expression of some suppressor genes to improve patient prognosis, thus antagonizing the effect of oncogenes. If the tumors are all high expression of oncogenes and no feedback suppression of oncogenes, the patient’s OS will be significantly shorter. For example, overexpression of tumor suppressor lncRNA MEG3 in some cancer cell lines, including lung, squamous cell, and gastrointestinal cancers, prevented cell proliferation and induced apoptosis, thus contributing to patient prognosis (33). In this study, AC104088.1 and DLX6-AS1 may feedback suppress the poor prognostic effect of AL513318.2 by regulating SLC6A8 or other suppressor genes expression, however, this speculation needs to be further verified experimentally. Second, studies have demonstrated that some genes promote the development of cancer but inhibit its progression. The tumor microenvironment is a complex environment and the gene function is multifaceted. P53 was previously thought to inhibit cancer progression, and recent studies have found that he can promote cancer (34, 35). Mengjie Liu et al. performed an immunohistochemical staining study on colon cancer patient samples and revealed that high CXCL11 expression in colon cancer was associated with poor prognosis and could be used as a biomarker for prognosis (36). However, in terms of tumor progression Yingying Cao et al. found that CXCL11 could promote anti-tumor immunity to prolong survival (37). Therefore, tumorigenesis and progression are not always interrelated. In this study, high expression of AC104088.1 and DLX6-AS1 in NSCLC exhibited good prognosis. However, the ceRNA regulatory network we constructed showed that these three lncRNAs targeting the downstream molecule hsa-miR-26a-5p/SLC6A8 were associated with poor prognosis. Finally, from a prognostic marker perspective, it does not affect AL513318.2 as a biomarker of poor prognosis in NSCLC. AL513318.2-targeted downstream molecule hsa-miR-26a-5p/SLC6A8 regulates patient’s final state also predicting poor prognosis. Therefore, DLX6-AS1 and AC104088.1 do not affect AL513318.2 as a biomarker of poor prognosis. It has been demonstrated that the different biological functions of lncRNAs depend mainly on their different subcellular localizations (38). Nuclear lncRNAs are rich in functions involving chromatin interactions, transcriptional regulation and RNA processing, while cytoplasmic lncRNAs can regulate mRNA stability or translation and influence cellular signaling cascades (10, 39, 40). Our study shows that AL513318.2 is mainly located in the cytoplasm and nucleus. Therefore it is essential for stable transcription and translation of *SLC6A8*. In addition, methylation analysis revealed that the cg12177562, cg13762255 and cg20765985 sites were significantly negatively correlated with *SLC6A8* expression, and survival analysis indicated that the low expression group of these sites had a poorer prognosis compared to the high expression group. This implies that cg12177562, cg13762255 and cg20765985 may be targets that regulate poor prognosis of *SLC6A8*, however the relationship of these sites with AL513318.2 and hsa-miR-26a-5p in the ceRNA regulatory network is unclear and requires further experimental analysis.

Recent studies revealed that the *SLC6A8*-encoded creatine transporter protein can deplete intracellular creatine altering macrophage-mediated immune responses *in vivo* (41). In addition, Stefano Di Biase et al. also reported that *SLC6A8*-mediated creatine transport is an important metabolic regulator of T-cell immunity (42). In this article, we confirmed the relevance of *SLC6A8* to the immune microenvironment of NSCLC in terms of immune cell surface marker genes and immunomodulators. Immunomodulators play an important role in the tumor microenvironment and are closely associated with patient immunotherapy and prognosis (24, 25). An immune prognostic risk model was constructed based on prognosis-related immunomodulators. The results showed that the high-risk group had a poorer prognosis than the low-risk group. Moreover, the risk scoring of the model can be used as an independent clinical prognostic risk factor. These studies demonstrated from the immunological aspect that *SLC6A8*, a target of the ceRNA regulatory network, is associated with poor prognosis in NSCLC. Finally, the interesting finding was that immunotherapy had a better treatment effect in the high-risk group than in the low-risk group. We speculate that this may be related to *SLC6A8*-mediated immune alterations. However, the exact cause needs to be further investigated.

In summary, AL513318.2 acts as a ceRNA network competing for binding to hsa-miR-26a-5p to regulate high expression of *SLC6A8* in NSCLC and is associated with poor prognosis. Therefore, the AL513318.2/hsa-miR-26a-5p/*SLC6A8* regulatory network may serve as a novel prognostic biomarker and potential therapeutic target for NSCLC treatment.

## Data Availability Statement

The original contributions presented in the study are included in the article/**Supplementary Material**. Further inquiries can be directed to the corresponding authors.

## Ethics Statement

The studies involving human participants were reviewed and approved by Changzhou No. 2 People’s Hospital Medical Ethics Committee. The patients/participants provided their written informed consent to participate in this study.

## Author Contributions

This subject and manuscript were designed and written by YF and YZ. The experiment was completed by YF. Data compilation was analyzed by XL. ML and ZG were responsible for the literature search to improve the project and revise the manuscript. All authors reviewed the manuscript and approved the manuscript for publication.

## Funding

This work is supported by the following funds: “333 Project” of Jiangsu Province (Grant number: BRA2020157); “Six One Project”, Research Projects of High-level Medical Personnel of Jiangsu Province (Grant number: LGY2019025); High-level Talent Selection and Training Project of the 16th Batch of “Six Talent Peak” in Jiangsu Province (Grant number: WSN-245); Medical Scientific Research Foundation of Jiangsu Commission of Health (Grant number: H2018083); Jiangsu Provincial Medical Youth Talent (Jiangsu Health Scientific Education 2017 no.3); 333 High-Level Talent Training Project (Grant number: 2016, III-0719); High-Level Medical Talents Training Project (Grant number: 2016CZBJ042).

## Conflict of Interest

The authors declare that the research was conducted in the absence of any commercial or financial relationships that could be construed as a potential conflict of interest.

## Publisher’s Note

All claims expressed in this article are solely those of the authors and do not necessarily represent those of their affiliated organizations, or those of the publisher, the editors and the reviewers. Any product that may be evaluated in this article, or claim that may be made by its manufacturer, is not guaranteed or endorsed by the publisher.

## Abbreviations

ceRNA, competitive endogenous RNA; lncRNA, long non-coding RNA; miRNA, microRNA; NSCLC, non-small cell lung cancer; LUAD, lung adenocarcinoma; LUSC, lung squamous cell carcinoma; TCGA, the Cancer Genome Atlas; TMA, tissue microarrays; AOD, average optical density; TIDE, Tumor Immune Dysfunction and Exclusion; LogFC, log_2_FoldChange.
