# Corrigendum: Long Non-Coding RNA AL513318.2 as ceRNA Binding to hsa-miR-26a-5p Upregulates *SLC6A8* Expression and Predicts Poor Prognosis in Non-Small Lung Cancer

**DOI:** 10.3389/fonc.2022.878864

**Published:** 2022-03-15

**Authors:** Yongfei Fan, Yong Zhou, Xinwei Li, Ming Lou, Zhaojia Gao, Kai Yuan, Jichun Tong

**Affiliations:** ^1^ Department of Thoracic Surgery, The Affiliated Changzhou No. 2 People’s Hospital of Nanjing Medical University, Changzhou, China; ^2^ Department of Gastroenterology, Affiliated Cancer Hospital of Bengbu Medical College, Bengbu, China; ^3^ Heart and Lung Disease Laboratory, The Affiliated Changzhou No. 2 People’s Hospital of Nanjing Medical University, Changzhou, China

**Keywords:** competitive endogenous RNA (ceRNA), *SLC6A8*, AL513318.2, hsa-miR-26a-5p, non-small cell lung cancer (NSCLC), prognosis

In the published article, there was an error in the order of the listed authors. The correct author list appears above.

The authors apologize for this error and state that this does not change the scientific conclusions of the article in any way. The original article has been updated.

## Publisher’s Note

All claims expressed in this article are solely those of the authors and do not necessarily represent those of their affiliated organizations, or those of the publisher, the editors and the reviewers. Any product that may be evaluated in this article, or claim that may be made by its manufacturer, is not guaranteed or endorsed by the publisher.

