# Supplementary figures and images for: Long Non-Coding RNA AL513318.2 as ceRNA Binding to hsa-miR-26a-5p Upregulates SLC6A8 Expression and Predicts Poor Prognosis in Non-Small Lung Cancer
Source: Front Oncol. 2022 Feb 16;12:781903. doi: 10.3389/fonc.2022.781903 (PMC8892383; doi:10.3389/fonc.2022.781903)

DNA methylation changes with SLC6A8 expression in LUAD

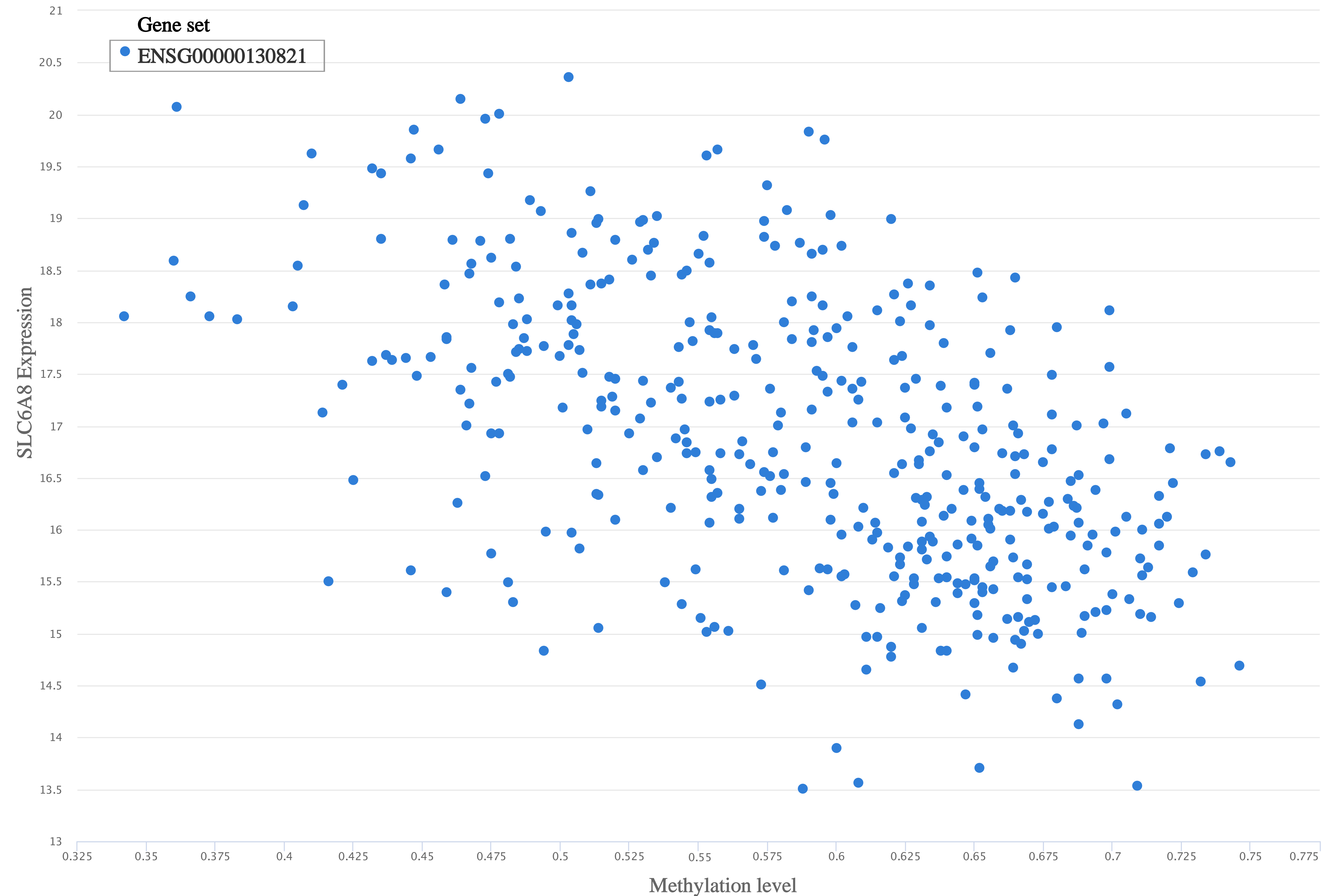

Supplement: Supplementary file 1 [file DataSheet_1.pdf]

DNA methylation changes with SLC6A8 expression in LUSC

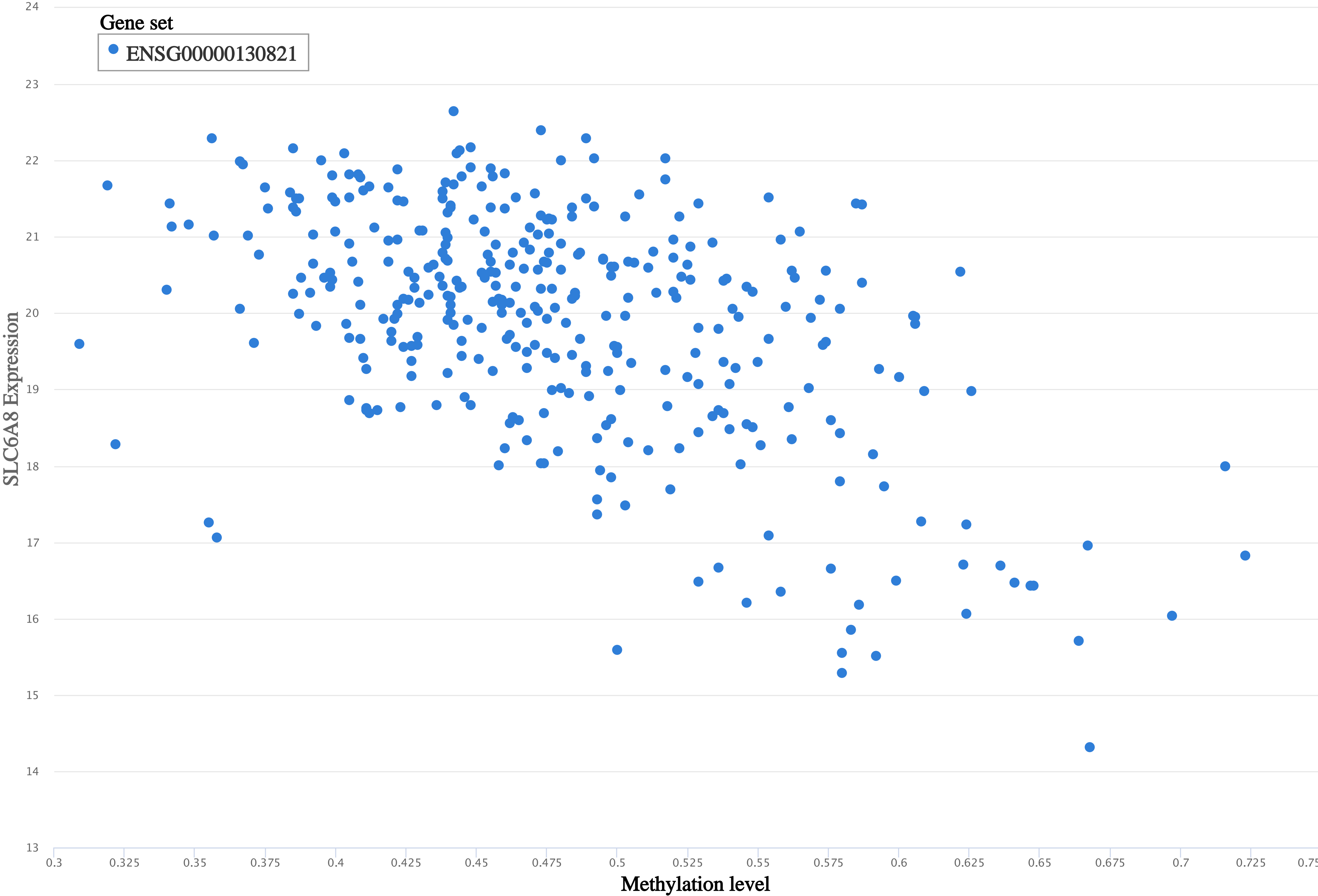

Supplement: Supplementary file 2 [file DataSheet_2.pdf]
