# Supplementary material for: Long Non-Coding RNA AL513318.2 as ceRNA Binding to hsa-miR-26a-5p Upregulates SLC6A8 Expression and Predicts Poor Prognosis in Non-Small Lung Cancer
Source: Front Oncol. 2022 Feb 16;12:781903. doi: 10.3389/fonc.2022.781903 (PMC8892383; doi:10.3389/fonc.2022.781903)
Supplement: Supplementary file 3 [file DataSheet_3.pdf]

LUAD

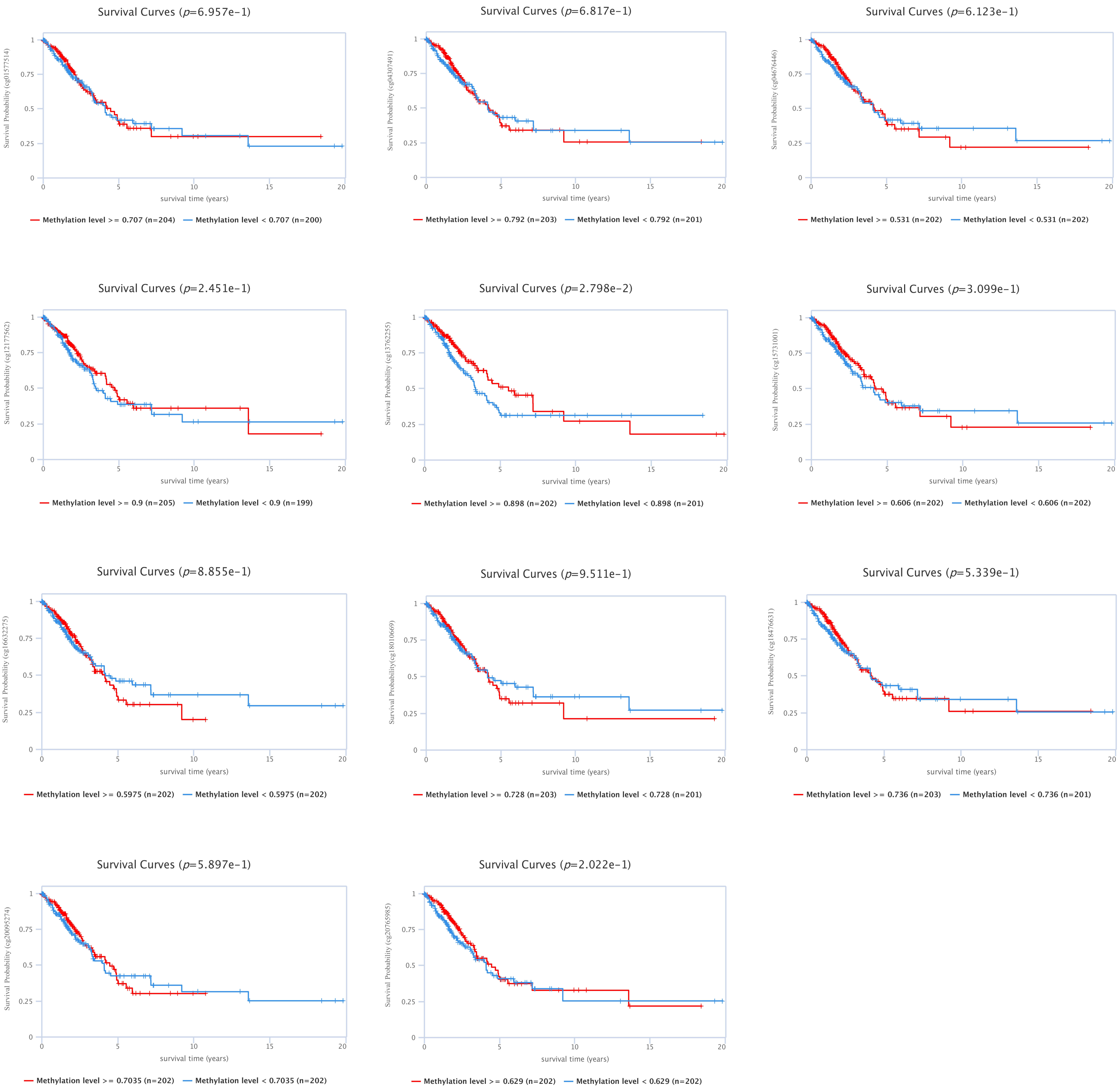

Survival Curves (p=6.957e-1)
Survival Probability (cg01577514)
survival time (years)
Methylation level >= 0.707 (n=204)
Methylation level < 0.707 (n=200)
Survival Curves (p=6.817e-1)
Survival Probability (cg04307491)
survival time (years)
Methylation level >= 0.792 (n=203)
Methylation level < 0.792 (n=201)
Survival Curves (p=6.123e-1)
Survival Probability (cg04676446)
survival time (years)
Methylation level >= 0.531 (n=202)
Methylation level < 0.531 (n=202)
Survival Curves (p=2.451e-1)
Survival Probability (cg12177562)
survival time (years)
Methylation level >= 0.9 (n=205)
Methylation level < 0.9 (n=199)
Survival Curves (p=2.798e-2)
Survival Probability (cg13762255)
survival time (years)
Methylation level >= 0.898 (n=202)
Methylation level < 0.898 (n=201)
Survival Curves (p=3.099e-1)
Survival Probability (cg15731001)
survival time (years)
Methylation level >= 0.606 (n=202)
Methylation level < 0.606 (n=202)
Survival Curves (p=8.855e-1)
Survival Probability (cg16632275)
survival time (years)
Methylation level >= 0.5975 (n=202)
Methylation level < 0.5975 (n=202)
Survival Curves (p=9.511e-1)
Survival Probability(cg18010669)
survival time (years)
Methylation level >= 0.728 (n=203)
Methylation level < 0.728 (n=201)
Survival Curves (p=5.339e-1)
Survival Probability (cg18476631)
survival time (years)
Methylation level >= 0.736 (n=203)
Methylation level < 0.736 (n=201)
Survival Curves (p=5.897e-1)
Survival Probability (cg20095274)
survival time (years)
Methylation level >= 0.7035 (n=202)
Methylation level < 0.7035 (n=202)
Survival Curves (p=2.022e-1)
Survival Probability (cg20765985)
survival time (years)
Methylation level >= 0.629 (n=202)
Methylation level < 0.629 (n=202)

LUSC

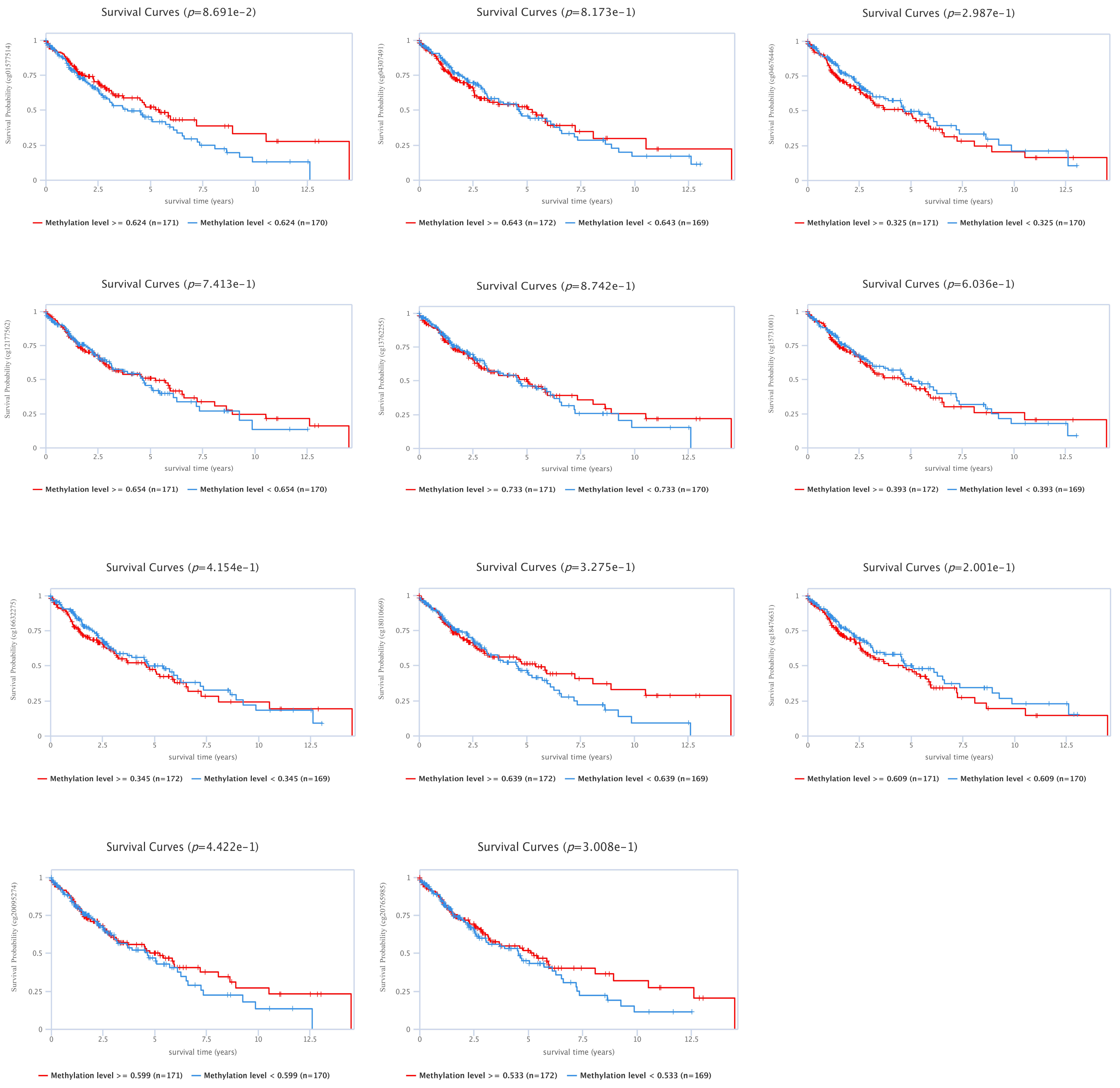

Survival Curves (p=8.691e-2)
Survival Probability (cg01577514)
survival time (years)
Methylation level >= 0.624 (n=171)
Methylation level < 0.624 (n=170)
Survival Curves (p=8.173e-1)
Survival Probability (cg04307491)
survival time (years)
Methylation level >= 0.643 (n=172)
Methylation level < 0.643 (n=169)
Survival Curves (p=2.987e-1)
Survival Probability (cg04676446)
survival time (years)
Methylation level >= 0.325 (n=171)
Methylation level < 0.325 (n=170)
Survival Curves (p=7.413e-1)
Survival Probability (cg12177562)
survival time (years)
Methylation level >= 0.654 (n=171)
Methylation level < 0.654 (n=170)
Survival Curves (p=8.742e-1)
Survival Probability (cg13762255)
survival time (years)
Methylation level >= 0.733 (n=171)
Methylation level < 0.733 (n=170)
Survival Curves (p=6.036e-1)
Survival Probability (cg15731001)
survival time (years)
Methylation level >= 0.393 (n=172)
Methylation level < 0.393 (n=169)
Survival Curves (p=4.154e-1)
Survival Probability (cg16632275)
survival time (years)
Methylation level >= 0.345 (n=172)
Methylation level < 0.345 (n=169)
Survival Curves (p=3.275e-1)
Survival Probability (cg18010669)
survival time (years)
Methylation level >= 0.639 (n=172)
Methylation level < 0.639 (n=169)
Survival Curves (p=2.001e-1)
Survival Probability (cg18476631)
survival time (years)
Methylation level >= 0.609 (n=171)
Methylation level < 0.609 (n=170)
Survival Curves (p=4.422e-1)
Survival Probability (cg20095274)
survival time (years)
Methylation level >= 0.599 (n=171)
Methylation level < 0.599 (n=170)
Survival Curves (p=3.008e-1)
Survival Probability (cg20765985)
survival time (years)
Methylation level >= 0.533 (n=172)
Methylation level < 0.533 (n=169)
